# Transverse gradient in Apple-type undulators

**DOI:** 10.1107/S1600577517004726

**Published:** 2017-04-07

**Authors:** M. Calvi, C. Camenzuli, E. Prat, Th. Schmidt

**Affiliations:** a Paul Scherrer Institute, CH-5232 Villigen PSI, Switzerland; b Department of Micro and Nanoelectronics, University of Malta, Msida, Malta

**Keywords:** soft X-ray line, Apple-type undulator, transverse gradient undulator

## Abstract

Apple-type undulators are globally recognized as the most flexible devices for the production of variable polarized light in the soft X-ray regime, both at synchrotron and free-electron laser facilities. Recently, the implementation of transverse gradient undulators has been proposed to enhance the performance of new generation light sources. In this paper it is demonstrated that Apple undulators do not only generate linear and elliptical polarized light but also variable transverse gradient under certain conditions. A general theoretical framework is introduced to evaluate the *K*-value and its transverse gradient for an Apple undulator, and formulas for all regular operational modes and different Apple types (including the most recent Delta type and Apple X) are calculated and critically discussed.

## Abstract 2

This theory demonstrates the existence of a variable transverse *K* gradient in Apple-type undulators. It is supported by numerical simulations to assess the limits of this analytical approach.

## 1. Introduction   

Since the late 1970s several insertion devices have been proposed to generate circular polarized light in the soft X-ray or UV regimes, both in synchrotron and free-electron laser (FEL) facilities. Although a comprehensive review of these devices is beyond the scope of this paper (Clarke, 2004 ▸), it is relevant to discuss in more detail the historical development of Apple (advanced planar polarization light emitter) undulators.

**Figure 1:**
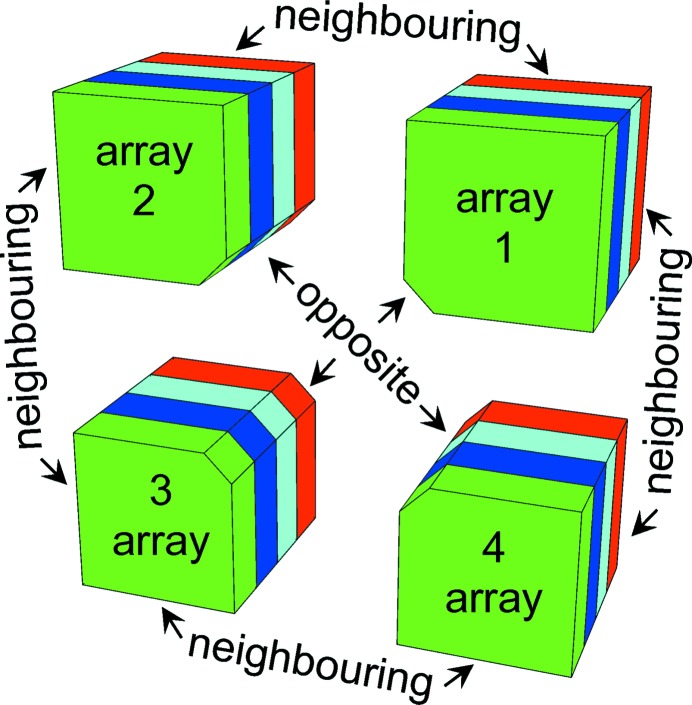
The four magnetic arrays of an Apple undulator are schematically represented to highlight the basic topology involved in this undulator concept: array pairs 1–2, 2–3, 3–4 and 4–1 are called neighbouring arrays, while pairs 1–3 and 2–4 are called opposite arrays.

The idea of the first Apple undulator (Sasaki *et al.*, 1993 ▸) was developed two decades later. Four magnetic arrays were implemented for the first time in a configuration which generates both linear and circular polarized light. As seen in Fig. 1 ▸, each array consists of a row of pure permanent magnets arranged in a Halbach magnetic configuration (Halbach, 1983 ▸) which generates a sinusoidal-like profile in both the *x*- and *y*-axes. Only two magnetic arrays standing opposite to each other are movable along the beam axis to switch from linear to circular polarization (*parallel mode*), and a *gap drive system* changes the distance between the upper and lower arrays to set the *K*-value.

**Figure 2:**
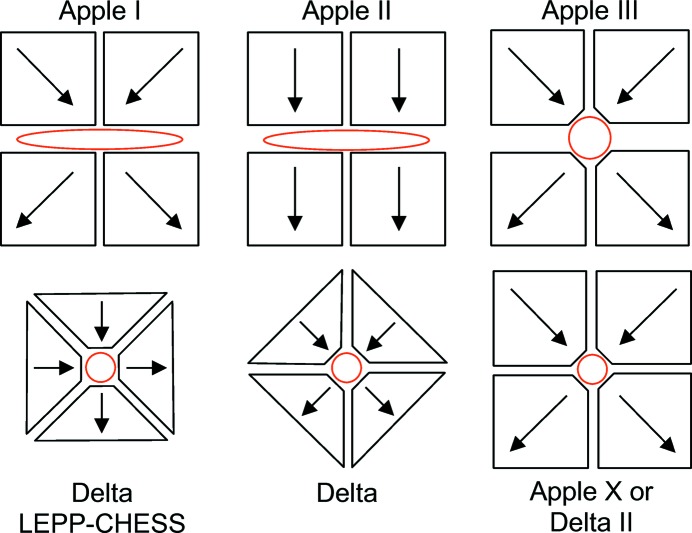
The different cross sections of Apple-type undulators in chronological order. In the first row one can find the standard Apple cross sections, starting with Apple I and II, which are designed for synchrotrons operated with elliptical vacuum chambers, followed by Apple III for linac-driven FELs. All three types are regularly equipped with a gap drive system which is changing the distance between the upper and lower arrays. In the second row there are the new cross sections called Delta LEPP-CHESS and Delta, which are designed for linac-driven FELs without a gap drive system. The last type is called Apple X (at the SwissFEL project) or Delta II (at the LCLS project), where a new gap drive system is moving an Apple III cross section in a way that both axis symmetries and 90° symmetry are guaranteed.

There are currently two other variants of this design called Apple II (Sasaki, 1994 ▸) and Apple III (Bahrdt *et al.*, 2004 ▸), having designs which differ in the magnet cross section (see Fig. 2 ▸). After the implementation of the first Apple II units, a new operational mode, now called *antiparallel mode*, was proposed in order to generate linearly polarized light with different angles, from linear horizontal to linear vertical polarization. This operational mode required a change in the design of the undulator support frame to be able to withstand the longitudinal forces that are present in this new operational mode but were negligible in the previous modes. The user community asked to further extend this mode to cover all angles (from 0° to 180°), leading finally to the introduction of four independent movable arrays along the beam axis.

Since an Apple undulator with four independent arrays no longer requires a gap drive system to set a different *K*-value (Carr, 1991 ▸), the implementation of novel devices was triggered. The first of these undulators was developed at the Swiss Light Source (Schmidt *et al.*, 2007 ▸): the device had an Apple II cross section, no gap drive system (*fixed gap*) and four independent arrays. Recently a new type of device, called Delta LEPP-CHESS due to the specific shape of its magnets (Temnykh, 2008 ▸), was proposed. Its cross section not only satisfies the usual axis (*x* and *y*) symmetry but also the 90° rotational symmetry which simplifies the operation. This device is based on the same operational principles as the previous fixed-gap undulator type but with a cross section rotated by 45°, as seen in Fig. 2 ▸. This device has now been adopted as an *afterburner* at the Linac Coherent Light Source (LCLS) facility but with the cross section rotated back to the original symmetry. It is now referred to as Delta (Nuhn *et al.*, 2015 ▸). In 2016 the Apple X was proposed at the Paul Scherrer Institute for the soft X-ray line of the SwissFEL. It consists of a Delta cross section where the four arrays can be independently displaced both longitudinally and radially. If the four arrays are displaced radially by the same amount, the 90° symmetry is preserved for all gaps. At the same time, it is also possible to displace them to break the symmetry, thus eventually introducing a gradient on-axis. The same development is ongoing at the LCLS and the device is referred as a Delta II (H.-D. Nuhn, private comunication).

### 1.1. Advantages and disadvantages of fix-gap operation   

The new operational mode, now called *energy mode*, is based on the parallel movement of two neighbouring arrays: the two top arrays (1 and 2) against the two bottom arrays (3 and 4) or the two left arrays (2 and 3) against the two right arrays (1 and 4), as illustrated in Fig. 1 ▸. The lack of a gap drive system to change the *K*-value increases cost effectiveness, while decreasing design complexity and the weight of the device. However, this comes with some drawbacks. The experimental evidence of these limitations was measured at the Swiss Light Source (Schmidt *et al.*, 2013 ▸) and was explained by the presence of a transversal *K* gradient. The resonance condition, expressed in equation (1) below, 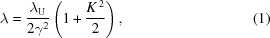
where 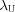 is the undulator period length and γ is the Lorenz factor, gives the relation between *K* and the radiation wavelength λ. In standard operation it is not desired that the radiation wavelength depends on the transverse position of the beam because it reduces the intensity of the interference peaks of the undulator spectrum. However, Schmidt’s work highlighted for the first time the possibility to operate an Apple undulator as a variable transverse gradient undulator (TGU).

Recently, many authors have demonstrated that TGUs may be useful for certain applications. They can be used to produce FEL radiation with large energy spread beams generated in laser-plasma accelerators (Huang *et al.*, 2012 ▸). If the electron energy is correlated to a transverse offset *via* dispersion and a TGU is set such that the resonance condition expressed in equation (1) is preserved for all the electrons, the performance of the FEL radiation will significantly improve.

A TGU can also be employed to generate ultra-large bandwidth radiation above the 10% level, which is needed for selected applications such as crystallography and spectroscopy (Prat *et al.*, 2016 ▸). This will occur when the beam is presented with a transverse tilt (correlation between the transverse and longitudinal positions of the electrons). Standard facilities use a stepwise tapering of the undulator field (Kroll *et al.*, 1981 ▸) (*i.e.* the undulator *K* is constant within an undulator module) to maximize the extracted FEL pulse energy. A TGU with variable gap can be used to generate a continuous taper within the undulator by transversely tilting the module. A continuous taper allows the extraction of more FEL pulse energy than a stepwise taper, which can only approximate the optimum taper along the undulator beamline. The continuous taper achievable with a TGU can be used to passively lock the FEL signal to an external laser signal (Saldin *et al.*, 2006 ▸). For the Athos beamline at SwissFEL, a continuous taper over an undulator modulator *via* a TGU is mandatory to achieve good performances since no significant contrast ratios can be achieved for a stepwise taper (Prat & Reiche, 2015 ▸).

In §2 and §3, a general and new theoretical framework is introduced to demonstrate the presence of a transverse gradient in an Apple undulator under certain operation conditions and to provide practical formulas for the actual operation of these devices. It is interesting to show that this analytical model also yields the same results published before (Schmidt & Zimoch, 2007 ▸). In addition, new conclusions can be derived, *e.g.* a simple relation between the energy shift and the transverse gradient in elliptical polarization, useful to tune the device for the new set of operations previously described.

## 2. Magnetic field model of an Apple undulator   

The purpose of this section is to estimate the magnetic field and its Jacobian in an Apple undulator knowing the field and the Jacobian of one magnetic array. There are several computer codes available to calculate the magnetic field produced by permanent magnets. In this paper, all magnetic computations are made with the *RADIA* code (Chubar *et al.*, 1998 ▸).

In an Apple undulator, the magnetic field can be approximated, with good accuracy, by the sum of the contributions of the four magnetic arrays, assuming that the permeability of the magnet material 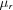 = 1. This is a reasonable assumption that can be made for NdFeB and SmCo magnets. Specifically, SmCo_5_ magnets have the lowest permeability (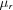 < 1.02) among these families of rare-earth materials and thus they are the first choice for these applications where the low field integral over the full operational range is specified and high remanence (above 1 T) is not required. Thus, the following mathematical description is traditionally the starting point for modelling the transverse magnetic field (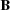 = 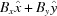) of all these devices, 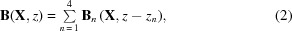
where 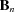 is the transversal magnetic field of the *n*th array, 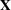 is the vector representing the cross-section plane (*xy*-plane), *z* is the coordinate along the beam axis and 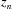 is the position of the *n*th magnetic array along the *z*-axis. The four arrays are identical (the field error contributions are not relevant for the purpose of this study and they are not discussed in this paper) and their relative positions follow a given symmetry. The magnetic field generated by each of the four arrays can be expressed using a linear transformation starting from one of the arrays. In equation (3) below, the first array is used for this purpose, 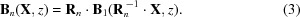
For standard Apple devices, the following matrices can be used to describe the relative position and symmetries among the arrays (the Delta LEPP-CHESS type does not directly follow this role but can easily be included in this theoretical framework as will be clarified later in §4.3), 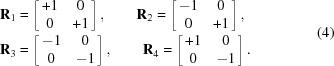
The next step in this analysis is the description of the *z*-axis in the Fourier domain. The field generated by the *n*th array transforms as follows, 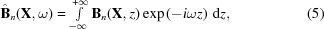
where the circumflex (^) indicates that the function is defined in the Fourier domain. There are two main advantages of adopting this formal description. The first advantage is related to the periodicity of the magnetic field along the *z*-axis of an undulator and its natural description of the Fourier domain. Moreover, it is usually enough to use the first harmonic to give an estimation of the field profile, thus reducing it to a single complex number (*i.e.* a phasor). The second advantage is related to the substitution of a translation into a product with a complex number.

Assuming a pure sinusoidal profile of the field along the *z*-axis with the periodicity of the undulator (the theoretical framework of this analysis can be extended to the full Fourier spectrum but it is beyond the purpose of the present publication), the *n*-array field profile can be simplified, 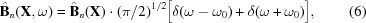
where the *xy*-plane dependence is factorized with the Fourier space part. Here, the same symbol is used for two different functions to simplify the notation since the ω dependence will not play an active role any longer. For the same reason, the δ functions will no longer be considered in the following equations, 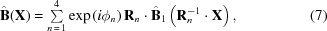
where the shifts in the *z*-axis are now substituted by four complex numbers with phase 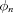 = 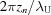. As a result of this approach, it is now simpler to express the Jacobian
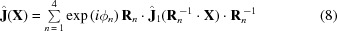
as a function of the Jacobian of the first-term array, 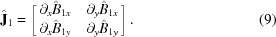
For the purpose of this publication, the main interest is focused on the field properties on the undulator axis, *i.e.* for 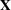 = 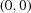. To further simplify the notation, all functions without explicit *xy*-plane dependence must be assumed to be evaluated on the axis from this point onwards. Following this rule, equations (7) and (8), respectively, become 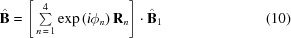
and 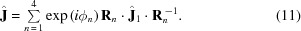
The components of 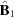 and 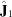 are assumed to be real numbers for the remainder of the paper. Since the average row phase is arbitrary, it will not affect the final results. Hence, it can be equated to zero for the sake of convenience.

Using the following change of variables, 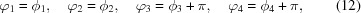
the linear horizontal polarization (pure vertical field) is recovered when 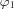 = 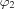 = 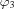 = 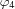 = 0, which is the configuration where all arrays are traditionally considered at zero mechanical position, *i.e.* zero shift position. Defining the following auxiliary 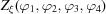 functions, 
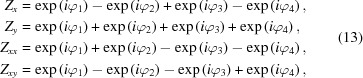
it is possible to write equations (10) and (11) in the following explicit forms, 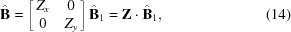


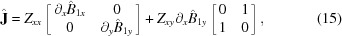
and for the highly symmetric case (relevant for later discussion), where 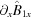 = 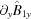, the Jacobian is simplified to the following expression,
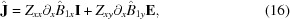
where 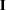 and 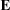 are, respectively, the identity and exchange matrix of rank 2. To summarize, there are four complex numbers which fully describe the status of the magnetic system in the neighbourhood of the undulator axis, and depend on the relative phase of the four arrays. Each 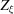 is the sum of four complex numbers 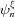 (note that 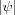 = 1) representing the status of the array, 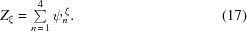
As a bookkeeping device, it is convenient to define the following matrix, 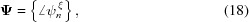
where the phase of each complex number is explicitly saved. To be more specific, an application for equation (18) for linear horizontal polarization is shown in equation (19) below,
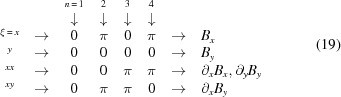
where each column represents one magnetic array and each row shows a specific property of the magnetic field. For instance, the first line represents the phases of the four complex numbers defining the *x* component of the magnetic field. In this matrix representation, a shift of a magnetic array is equivalent to the addition of the same phase to the column corresponding to the magnetic array. Following this pattern, the elliptical polarization (parallel operational mode, p) and the linear inclined polarization (antiparallel operational mode, 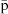) are, respectively, summarized by the following matrices, 
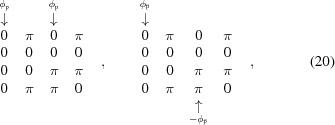
while the energy shift modes (mandatory modes for fixed-gap devices to set the *K*-value and change the photon energy) are represented by the following matrices, for the top–bottom mode (arrays 1 and 2) and for the left–right mode (arrays 2 and 3), respectively (as seen in Fig. 1 ▸), 
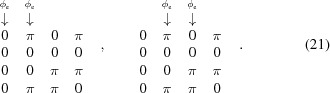
Knowing the magnetic field and its gradient is the first step towards a correct understanding of the electron dynamic along the undulator axis. A detailed study of the actual orbit is beyond the scope of this paper. During this investigation, the electrons are assumed to wiggle in a parallel and closed fashion (*i.e.* in the neighbourhood) to the undulator axis. In the following section, the undulator *K* and its gradient are estimated by starting with the results and assumptions presented in §2.

## 3. Estimation of the *K*-value and its gradient   

Defining κ as the coefficient to convert the magnetic field domain into the *K* domain, 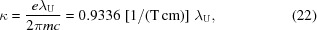
where 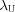 is the undulator period length, *e* and *m* represent the charge and the mass of the electron, respectively, and *c* defines the speed of light, it is practical to describe the vector 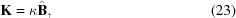
which simplifies the definition of *K* directly in the Fourier space (there is no need to transform back to the *z*-axis domain), 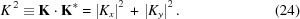
From equation (24) it is possible to estimate *K* for all operating modes as it is presented in §4. Continuing with the estimation of the *K* gradient, the first step is the differentiation of equation (24) both in *x* and *y* (for this intermediate step it is simpler to go back to *x* and *y* components separately), 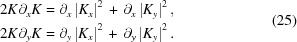
Re-using the definition of the complex conjugate (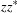 = 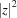), expression (25) above can be further simplified, giving
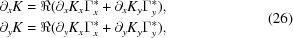
where 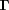 = 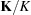. To further summarize this important result, it is possible to express it in matrix form in terms of the evaluated magnetic field. Noting that the Jacobian of 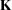 is proportional to the Jacobian of 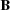, as is inferred from (11), the gradient of *K* can be finally expressed in the following compact form, 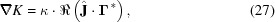
or explicitly as a function of the magnetic field, 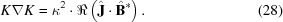
The right-hand side of equation (28) can be written as a function of the 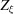 numbers, 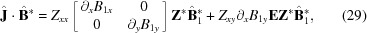
as well as *K*, 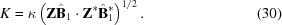
In the symmetric case where 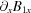 = 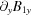, equation (29) simplifies to the following final expression, 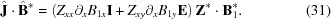



## 4. Analysis of the operational modes   

In this section, equations (24) and (27) are solved for the two operational modes: parallel (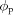) and antiparallel (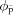). In both cases, the equations are directly derived by also assuming an energy shift (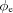). The domain of 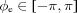 is restricted to simplify the formulas and to guarantee that all functions are analytical, especially the gradient. From equations (24) and (27) it is effortless to demonstrate that *K* and its gradient are invariant with respect to an arbitrary phase, as it should be for this calculation to become consistent with the evidence that the properties of the undulator do not depend on its longitudinal location. Subsequently, this result will also be used to simplify the phase shift definition, even when the shift produces a net displacement of the undulator structure. On the contrary, in a device which is installed on a beamline, it is important to keep its longitudinal position fixed, in order to prevent the actual source point from moving.

### 4.1. Apple I, II and III   

In standard Apple undulators (I, II, III), only the symmetry expressed in equation (4) is satisfied and usually 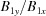 = 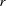 > 1. Solving for equation (24) for the parallel operational mode (elliptical light) using the following phase values for 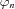, 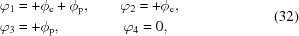
the *K*-value is equal to 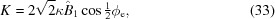
where 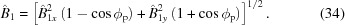
The *K* gradient can be obtained by solving equation (27). Equation (35) shows the results for top–bottom shift, 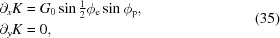
where 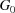 = 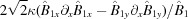, while equation (36) shows the results for the left–right shift, 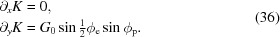
The relative variation of *K* with respect to *K* is the actual relevant parameter for some applications, 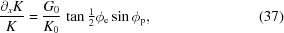
where 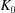 = 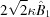. The condition to move from elliptical polarization to circular polarization is 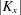 = 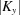, which corresponds to a phase 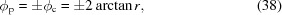
where the positive sign (+) indicates the anticlockwise circular polarization and the negative sign (−) shows its clockwise counterpart, looking in the direction of the electron. For the linear inclined mode, the phase is defined by 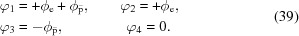
Using equation (24), the *K*-value is given by
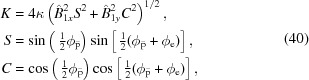
and the angle of the magnetic field, defined as 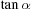 = 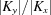, is calculated in equation (41) below, 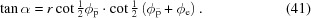
When 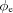 = 0, equation (40) simplifies to the more familiar result
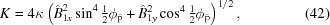


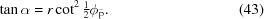
As a corollary of this general description, it is possible to recover the results (Schmidt & Zimoch, 2007 ▸) referred to as *symmetry phase*. In equation (44) below, the condition of *K* is independent of 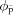, 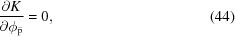
leading to the following interesting result, 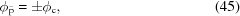


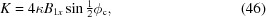


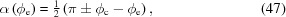
where the angle of polarization can be linearly varied with the former *energy shift* as a function of 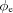 while keeping *K* constant. This special mode naturally requires a gap drive system to change the photon energy (*K*).

### 4.2. Apple X (Delta II) and Delta   

In an Apple X undulator, 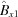 = 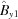 and 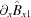 = 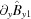 for all gaps. This is due to the radial displacement of the magnetic array, as seen in Fig. 2 ▸. This undulator cross section not only respects the symmetries to the main axis as all standard Apple undulators do, but also the symmetry with respect to a 90° rotation. This geometry simplifies all the results obtained in §3. It can be proven that the phase of the circular polarization 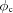 is gap independent and equal to 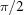, thus there is no need to correct the parallel shift position to recover the condition: 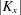 = 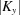. This simplifies the operation of the device, both in terms of modelling and manipulation, which should also help to reduce the ageing of the mechanical parts. The *K*-value now depends only on the gap and energy shifts and can be expressed as shown in equation (48) below, 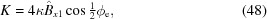
where 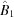 simplifies to 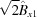 which depends only on the gap. Equation (48) does not depend on the parallel shift 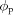 any longer. Therefore, the *K*-value for a given gap is the same for all elliptical configurations (including the special case of circular polarization) and thus substantially simplifies the operation of the device. The *K* gradient maintains the same formal expression as in equations (35), (36) and (37) while 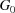 and 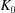 simplify as shown in the equations below, 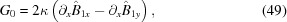


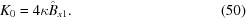
These parameters are no longer dependent on 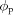, but are now only dependent on the gap. It is therefore possible to further simplify the previous expressions of the *K* gradient for circular polarization (specifically for 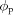 = π/2) and to explicitly write them as a function of *K*, 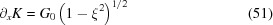
and 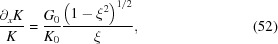
where 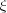 = 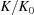. When 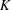 = 0 the gradient is maximized to the value 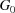.

For the inclined mode, equation (40) simplifies to the following expression, 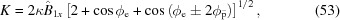
where the positive sign (+) corresponds to the top–bottom energy shift and the negative sign (−) corresponds to the left–right energy shift. As found in the general case, there are no *K* gradients for linear polarization of Apple X. All the results presented in this section also hold for the Delta undulator type, with the unique distinction that in this design no system is available to set the gap.

### 4.3. Delta LEPP-CHESS   

To analyse the Delta LEPP-CHESS type undulator, the symmetry seen in equation (4) is no longer valid. On the contrary, this cross section follows a symmetry with respect to the 45° axes. To study this cross section, it is possible to use a rotational symmetry in steps of 90° in place of equation (4), 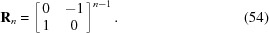
Following the analysis defined in §2 and §3, it is then possible to estimate the field and the gradient. Instead of applying this methodology, the previously calculated 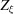 functions can be used directly after applying a simple axis rotation of 45°, 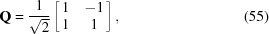
to equation (10) and to equation (11), respectively, 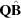 and 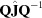. This approach also allows the use of the previously calculated magnetic field and Jacobian in the original reference frame and their properties: 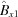 = 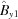 and 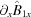 = 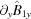. Therefore, the main results of this calculation are expressed as follows, 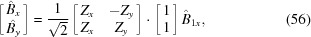


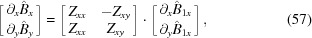


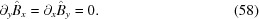
The *K*-value for the parallel mode is identical to the one evaluated for the Apple X case and is repeated for completeness’ sake in equation (59) below, 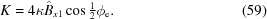
The *K* gradient, on the contrary, is present simultaneously in both planes as calculated in equation (60) below, 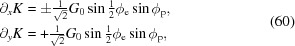
where the positive sign (+) represents the top–bottom energy shift and the negative sign (−) stands for the left–right energy shift. This is the major difference between this device and all other devices that are analysed in this paper. Its implementation in a facility has to be carefully evaluated by also taking into account the result of equation (60).

For the antiparallel mode, the results follow the general rule: no *K* gradient is present. The expression of *K* is very similar to that observed for Apple X [equation (53)], except for a change in sign. No change in signs is seen for the top–bottom and the left–right shifts, as shown in equation (61) below, 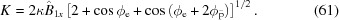



## 5. Model *versus* simulation for the Apple X   

**Figure 3:**
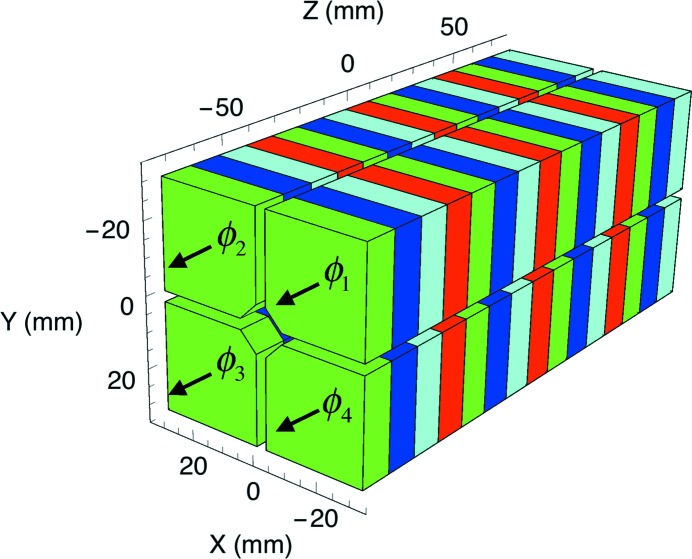
Magnetic model of the Apple X undulator used for the example. The actual model consists of eight periods while in this picture only four have been drawn.

The transversal gradient of an Apple X undulator has been evaluated with the help of the computer code *RADIA* to verify the quality of the analytical approach presented in this article and to highlight its limitations. The formulas derived for *K* have already been proven by other authors and are widely supported by experimental results. Thus, only the results concerning the transverse gradient are reported in this section. The geometry and the material properties of the magnetic structure are presented in Fig. 3 ▸ and in Table 1 ▸, respectively.

**Figure 4:**
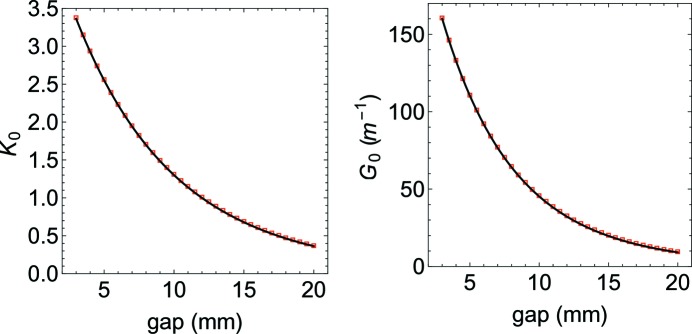
The values of 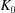 and 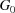 have been calculated as a function of the undulator (Apple X) gap. The numerical calculation (red markers) are presented together with the fitting functions (solid black line) introduced in equation (62).

**Figure 5:**
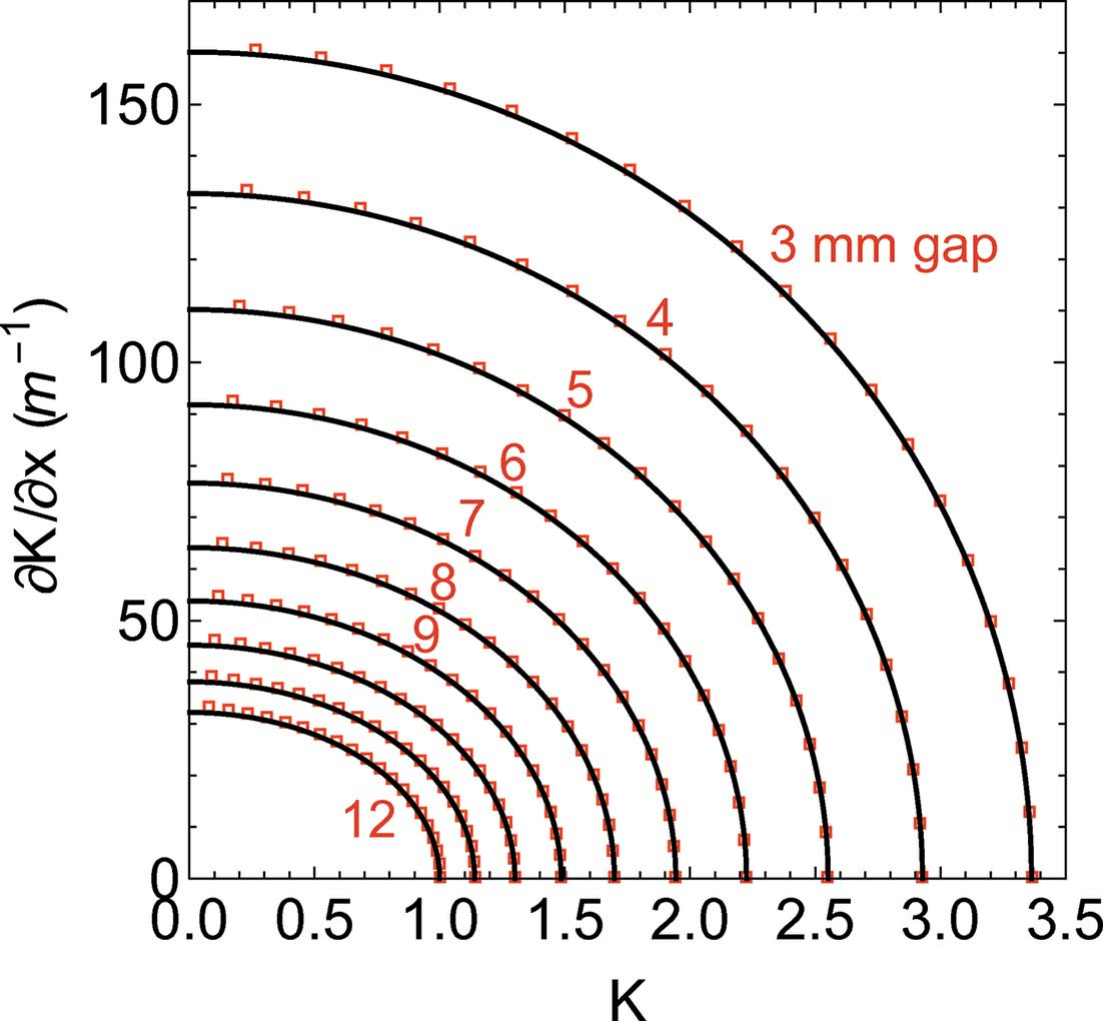
Horizontal component of the *K* gradient *versus* *K* for different gaps. The analytical model (solid line) is presented together with the computer simulation made with *RADIA* (red square markers).

**Figure 6:**
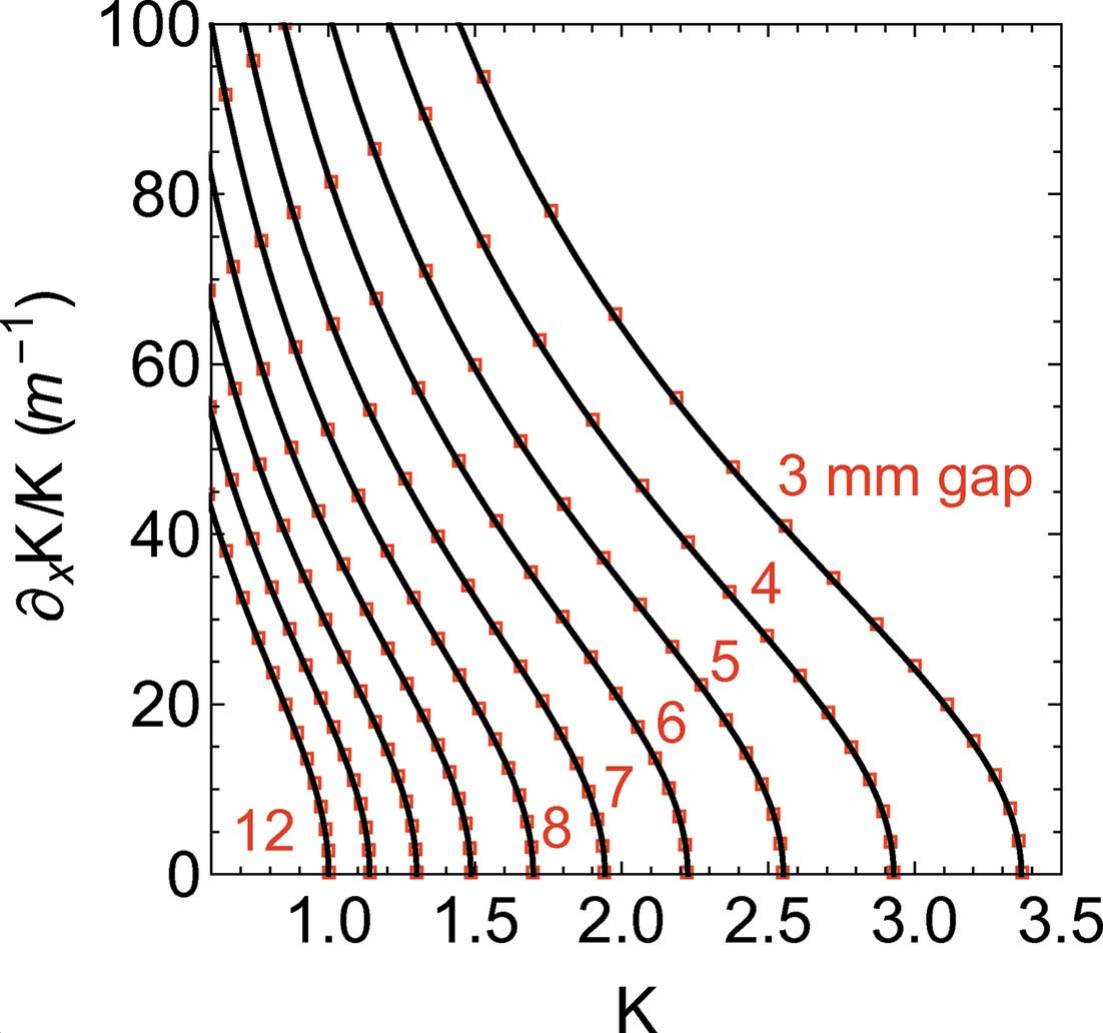
Horizontal component of the *K* gradient relative to *K* *versus* *K* for different gaps. The analytical model (solid line) is presented together with the computer simulation made with *RADIA* (red square markers).

To evaluate equation (51), the first step is to numerically calculate 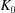 and 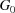 as formulated in equations (50) and (49), respectively (see Fig. 4 ▸). The results of both calculations are fitted with the following function (used for the *K* *versus* gap of a hybrid magnetic structure), 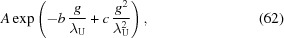
where the independent variable *g* is the gap. In Table 2 ▸ the coefficients are listed for both 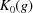 and 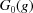. In Fig. 5 ▸, equation (51) is estimated both analytically (solid line) and numerically (markers). For completeness’ sake, equation (52) is presented in Fig. 6 ▸. There is a very good agreement between the analytical model and the numerical calculation, and the small deviations (the analytical approximation underestimates the gradient) are due mainly to the nonlinearity of the magnetic material which has been neglected and not to the single harmonic approximation.

It is also important to study the behaviour of the function in the neighbourhood of 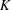 = 0. From the definition of *K* in equation (24), it is already clear that the gradient must be zero when *K* is zero and a smooth transition is expected while approaching this value but this is not present in the analytical approximation.

**Figure 7:**
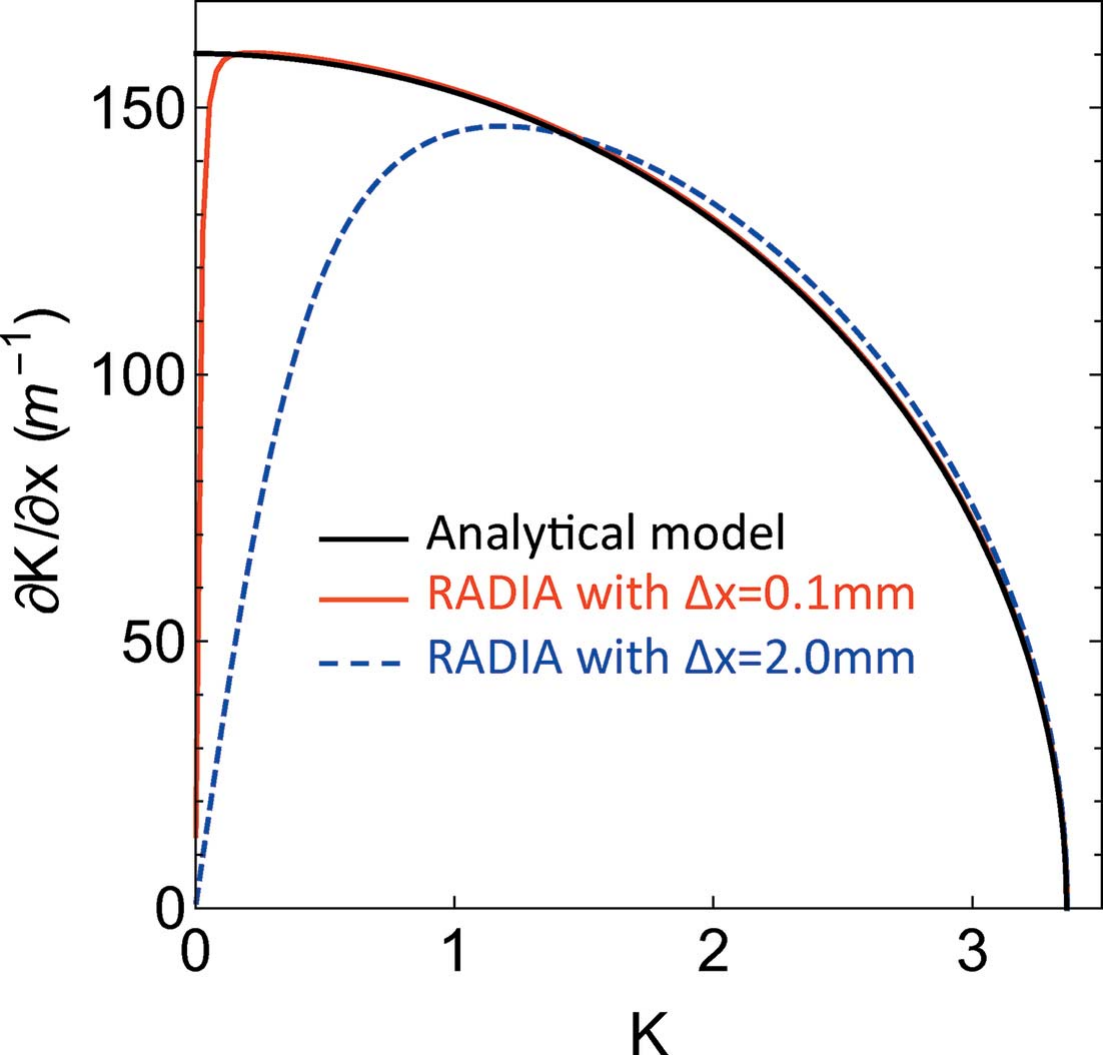
The horizontal component of the *K* gradient *versus* *K* is presented for a gap of 3.0 mm for both the analytical model (solid black line) and the *RADIA* simulations. Two numerical results are presented, one (solid red line) calculates the derivative using 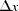 = 0.1 mm, the other (dashed blue line) using 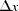 = 2.0 mm.

In Fig. 7 ▸, a simulation is presented for a gap of 3.0 mm with a finer mesh of *K*. Depending on the definition of the numerical derivative (in the specific example the calculations are made with 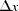 equal to 0.1 mm and 2.0 mm), it is possible to estimate the transition to zero. For the applications it is important to estimate the extension of the linear region where the gradient is correctly approximating the field profile. From the example there is a clear difference between the region of 0.1 mm, where the analytical approximation very accurately describes the value of gradient almost down to 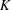 = 0, and the region of 2 mm, where the analytical approximation already fails below 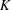 = 1.

## 6. Conclusions   

In Apple undulators it is mandatory to implement a gap drive system to decouple *K* from its gradient. The possibility to independently introduce a *K* gradient both in the *x*- and in the *y*-plane gives rise to the possibility of developing novel operating modes in synchrotron and FEL facilities. The implementation of fixed-gap devices has to be evaluated to fit the facility requirements. While the reduced costs and simplified logistics (due to the reduced weight) are attractive options, the coupling between *K* and 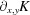 might be a serious issue. Apple X (Delta II) undulators improve the operation of the insertion device due to the higher degree of symmetry for any *K*. These undulators feature the unique property of controlled asymmetries (as far as each array has to be independently displaced in the radial direction) which can be used to introduce gradients in any polarization, which, for standard Apple devices, is limited to elliptical polarization. The details of this operation and the calculation of the scaling laws nevertheless require further studies and can be the subject of a new article.

## Figures and Tables

**Table 1:** Simulation parameters for the Apple X example

Parameter		Unit
λ_U_	40.0	mm
Magnet material	Sm_2_Co_17_	–
Remanence	1.08	T
Number of periods	8	–
Magnet edge	30.0	mm
Magnet chamfer	5.0	mm

**Table 2:** Fitting parameters for the analytical gradient estimation

	*A*	*b*	*c*
*K*_0_	5.1295	5.6845	0.7821
*G*_0_	285.42 m^−1^	7.8497	1.9248
